# Cognitive map formation through tactile map navigation in visually impaired and sighted persons

**DOI:** 10.1038/s41598-022-15858-4

**Published:** 2022-07-07

**Authors:** Loes Ottink, Bram van Raalte, Christian F. Doeller, Thea M. Van der Geest, Richard J. A. Van Wezel

**Affiliations:** 1 Donders Institute, Radboud University, Heijendaalseweg 135, P.O. Box 9010, 6500 GL Nijmegen, The Netherlands; 2 Psychology Department, Max Planck Institute for Human Cognitive and Brain Sciences, Leipzig, Germany; 3 Kavli Insitute for Systems Neuroscience, NTNU, Trondheim, Norway; 4 Lectorate Media Design, HAN University of Applied Sciences, Arnhem, The Netherlands; 5 Techmed Centre, Biomedical Signals and System, University of Twente, Enschede, The Netherlands

**Keywords:** Cognitive neuroscience, Learning and memory, Spatial memory

## Abstract

The human brain can form cognitive maps of a spatial environment, which can support wayfinding. In this study, we investigated cognitive map formation of an environment presented in the tactile modality, in visually impaired and sighted persons. In addition, we assessed the acquisition of route and survey knowledge. Ten persons with a visual impairment (PVIs) and ten sighted control participants learned a tactile map of a city-like environment. The map included five marked locations associated with different items. Participants subsequently estimated distances between item pairs, performed a direction pointing task, reproduced routes between items and recalled item locations. In addition, we conducted questionnaires to assess general navigational abilities and the use of route or survey strategies. Overall, participants in both groups performed well on the spatial tasks. Our results did not show differences in performance between PVIs and sighted persons, indicating that both groups formed an equally accurate cognitive map. Furthermore, we found that the groups generally used similar navigational strategies, which correlated with performance on some of the tasks, and acquired similar and accurate route and survey knowledge. We therefore suggest that PVIs are able to employ a route as well as survey strategy if they have the opportunity to access route-like as well as map-like information such as on a tactile map.

## Introduction

The brain can form a representation of the spatial environment around us^1,2^. Such a cognitive map can support navigation in this environment, and is essential for finding our way in familiar and unfamiliar environments. Cognitive map formation has been suggested since the early 1900s as imaginary maps^3^, and became more established by Tolman^4^. It has been studied further in rodents^2,5,6^, and subsequently in humans^1,7,8^. A cognitive map as considered in this study, incorporates locations, directions and distances between locations^7–10^. Having such a cognitive map can furthermore allow for a route-like (egocentric) as well as a survey-like (map-like, allocentric) representation of an environment^1,11^. While both route and survey representations are beneficial, a survey representation is more flexible. It allows a person to infer information that is not explicitly learned, such as Euclidean distances, detours and shortcuts between locations^12^. A cognitive map, as we consider it, can be built up through direct navigation; but it can also be a representation of a virtual environment or even a small-scale map^13^. In the current study, we wanted to contribute to the knowledge of mental representations of a small-scale environment. Specifically, we addressed cognitive map formation of a tactile map in persons with a visual impairment (PVIs) and sighted controls.

Most knowledge about cognitive maps is based on visual information. Less is known about this process when visual input is reduced or not available, for example in PVIs. These individuals rely mostly on tactile, haptic, auditory and proprioceptive information. Wayfinding is one of the main problems PVIs encounter during their daily life^14,15^. Many PVIs experience difficulties regarding autonomous navigation, causing them to travel less frequently. This affects their life on a personal and professional level, and can lead to societal exclusion^16,17^. Training in orientation and mobility can improve autonomous navigation by PVIs^18,19^. The orientation component, however, is often found to be difficult when a person relies on non-visual sensory information, possibly because vision conveys more spatial information at once compared to other sensory modalities^20^. Orientation involves processes such as spatial updating, mental rotation, finding shortcuts and computing detours, which are also related to cognitive map formation. These processes, however, are often, but not always, reported to be challenging without visual information^18–24^. Therefore, it is relevant to address cognitive map formation based on non-visual sensory information. In the current study, we focused on the tactile sensory modality. Several studies suggest that PVIs can form a cognitive map via tactile or haptic information, however, because they mostly rely on route knowledge^25–28^, their cognitive maps are somewhat less accurate or detailed than those of sighted persons^13,29–32^. For instance, blindfolded sighted participants make fewer errors on solving tactile mazes^13^, and cognitive maps of PVIs are less accurately scaled than those of sighted persons^29^. Additionally, especially early blind persons have less accurate mental representations of specific locations^21,30^, distances^31^, and directions^20,32^ of a tactile map compared to late blind and to sighted persons. Nevertheless, after extensive training, PVIs and sighted persons form equally accurate mental representations of for instance tactile map layout^33,34^ and locations^20,35^. Furthermore, numerous studies indicate the potency of tactile maps to support PVIs in orientation and wayfinding^25,29,36,37^. Purely tactile maps, such as raised-line paper or 3D-printed maps are useful in acquiring spatial knowledge of a building or outside environment^16,17^. Furthermore, interactive maps, which often combine tactile or haptic information with sound, provides for even more spatial information, enhancing spatial cognition in PVIs^17,38,39^. Interestingly, exploring tactile maps might even be more effective in giving PVIs an impression of an environment than direct experience^22,25,29^. Additionally, inspecting a tactile^17,40^ or audio-tactile map^17,27,38^ before navigating in the real environment improves wayfinding compared to real-world navigation only.

Cognitive map formation based on tactile information has been behaviourally assessed by addressing several spatial aspects, such as object location recall^21,35^, reproduction or recall of specific routes^13,25,36,40^, and reproduction of map configuration^29,34,41^. Additionally, estimation of distances^11,21,31,42^ or direction^11,20,32^ are also measures indicating the construction of a cognitive map by PVIs. These studies indicate that PVIs perform well on the various measures. Mental representations of small-scale tactile maps^21,31,32^ as well as real-world environments^11,20,42^ have been assessed. Most of this research, however, involved only a limited number of such spatial aspects. In the current study, we propose a more thorough overview of cognitive map formation based on identifying several key categories of spatial information and relationships.

Since we consider a cognitive map as allowing for a route as well as a survey representation, we further addressed the role of two main navigational strategies, route- and survey-type strategies. A route strategy relates to an egocentric (own body-centered) perspective, and focuses on a direct route between locations using a sequence of instructions^2,12^. A survey strategy relates to an allocentric (environment-centered, map-like) perspective, and centralises on a map overview and relations between locations^2,12^. The type of strategy people use during navigation, may be related to the type of mental spatial representations and the accuracy of cognitive maps^43^. Several studies suggest that PVIs mostly employ a route strategy and form a cognitive map that is mainly egocentric, because they are assumed to rely on egocentric information^20,28,44–46^. PVIs perform similar or better than sighted persons on tasks where they have to use a route representation^20,46^. Nonetheless, it has been shown that a survey representation can be present in PVIs as well, although they seem to face more difficulties constructing a survey than a route representation^28,33,44,45,47^. Some researchers also argue, however, that these difficulties arise because PVIs have less to no access to allocentric or survey information due to the way information is presented^27,28,48^. These studies suggest that when PVIs are offered opportunities to use allocentric information despite their impairment, they can employ this up until a similar level as sighted persons and form a survey representation. Because of the discrepancies in the literature about navigational strategies, we also addressed their role in the current study.

To assess cognitive map formation of a small-scale tactile city-like environment, we let sighted and visually impaired participants explore a tactile map including five item locations. Subsequently, participants performed several spatial tasks. These tasks allowed us to investigate whether they formed an accurate mental representation of the map and spatial relations between the item locations. First, they estimated relative Euclidean and path distances between each item pair in a distance estimation task. Subsequently, they performed a direction pointing task, reproduced routes between each item pair (route rebuilding task), and completed an item placement task where they recalled the item locations. The combination of tasks furthermore enabled us to assess route and survey representations. Good performance on the distance estimation task and pointing task most likely required a survey representation, whereas a route representation was presumably sufficient for the route rebuilding and item placement tasks. Furthermore, we wanted to investigate the relations between performance on the different spatial tasks and the navigational strategy our participants generally employ. To this end, they filled out the Santa Barbara Sense of Direction scale^49^ (SBSOD) about navigational abilities and sense of direction, and a Wayfinding Strategy Scale^12,50^ (WSS) about general use of navigational strategies.

We expected that all participants would perform well on the tasks that require a route representation. For the tasks that require a survey representation, we expected that sighted participants might perform slightly better than the PVIs, because blind persons often have difficulties constructing a survey representation^44,45^. We considered, however, that it is not visual experience but rather the tendency to use a survey or route strategy that relates to performance on these tasks^27,28,48^. Therefore, we think that possible relationships between the questionnaires and task performance will give more insight.

## Methods

### Participants

In total, 20 participants were included in this study. Ten participants were visually impaired (see Table 1 for details), and ten sighted participants formed a control group. These participants were matched to the PVI group on age, gender and education level. The sample size was calculated based on previous data and pilot experiments. All participants signed up for the experiment through advertisements or flyers distributed amongst client organisations and companies, fairs organised for PVIs, or through personal contacts of the participants and researchers.

**Table 1:** Details of visually impaired participants: age, gender, highest education level, visual impairment, age of onset, autonomous navigation, O&M training, and braille experience.

Age	Gender	Education level	Visual impairment	Age of onset	Autonomous navigation	O&M training	Braille experience
24	Female	University	Amalis congenita	Birth	Often	Yes	Yes
61	Male	PSVE	Usher syndrome	Birth	Often	No	No
72	Male	LSGE	Retinal detachment	41	Hardly	Yes	No
66	Male	University	Unknown	62	Regularly	Yes	Yes
55	Female	HVE	Rubella (of mother during pregnancy)	Birth	Often	No	No
30	Male	HVE	Retinal detachment	Birth	Often	Yes	Yes
69	Male	HVE	Aniridia	Birth	Regularly	No	Yes
71	Male	University	Macula degeneration	6	Often	Yes	No
66	Female	HVE	Rubella (of mother during pregnancy)	Birth	Regularly	Yes	Yes
68	Male	HVE	Rubella (of mother during pregnancy)	Birth	Often	Yes	Yes

*LSGE* lower secondary general education, *PSVE* post-secondary vocational education, *HVE* higher vocational education.

We only included PVIs with a visual impairment such that they were not able to read or use visual street maps for wayfinding. Additionally, none of the participants had cognitive or sensory impairments other than visual in the PVI group. Visual or hearing impairments corrected to normal were allowed for control participants. All participants were right-handed and Dutch-speaking.

During the experimental session, we conducted two questionnaires to evaluate general navigational abilities and strategies, the SBSOD and WSS. Additionally, we asked the PVIs about whether they often travel autonomously in their daily life, and about their experience in orientation and mobility (O&M) training and braille (see Table 1). Together, the results of all questionnaires indicated that most participants are relatively good navigators. The outcomes of the questionnaires are discussed in more detail in the “Results” section.

Ethical approval for the study was given via an amendment by the local ethical committee (CMO Regio Arnhem-Nijmegen, The Netherlands, nb. 2014/288). The experiments were performed in accordance with guidelines and regulations relevant for research involving human participants. All participants provided informed consent before the start of the experiment. Control participants gave written informed consent, and PVIs while being recorded by a video camera. These videos were stored in a different location from the rest of the experimental data.

During all tasks, all sighted control participants were blindfolded by a curtain in front of them. Only task-relevant visual information was taken away by this curtain, but participants could still look around the room. This was done because for sighted persons it would be an unusual and possibly uncomfortable situation to not have any visual input^23,51^. When PVIs had some residual vision, although very limited, we also used the curtain to ensure none of the participants could get any task-related visual input.

### Tactile map

**Figure 1:**
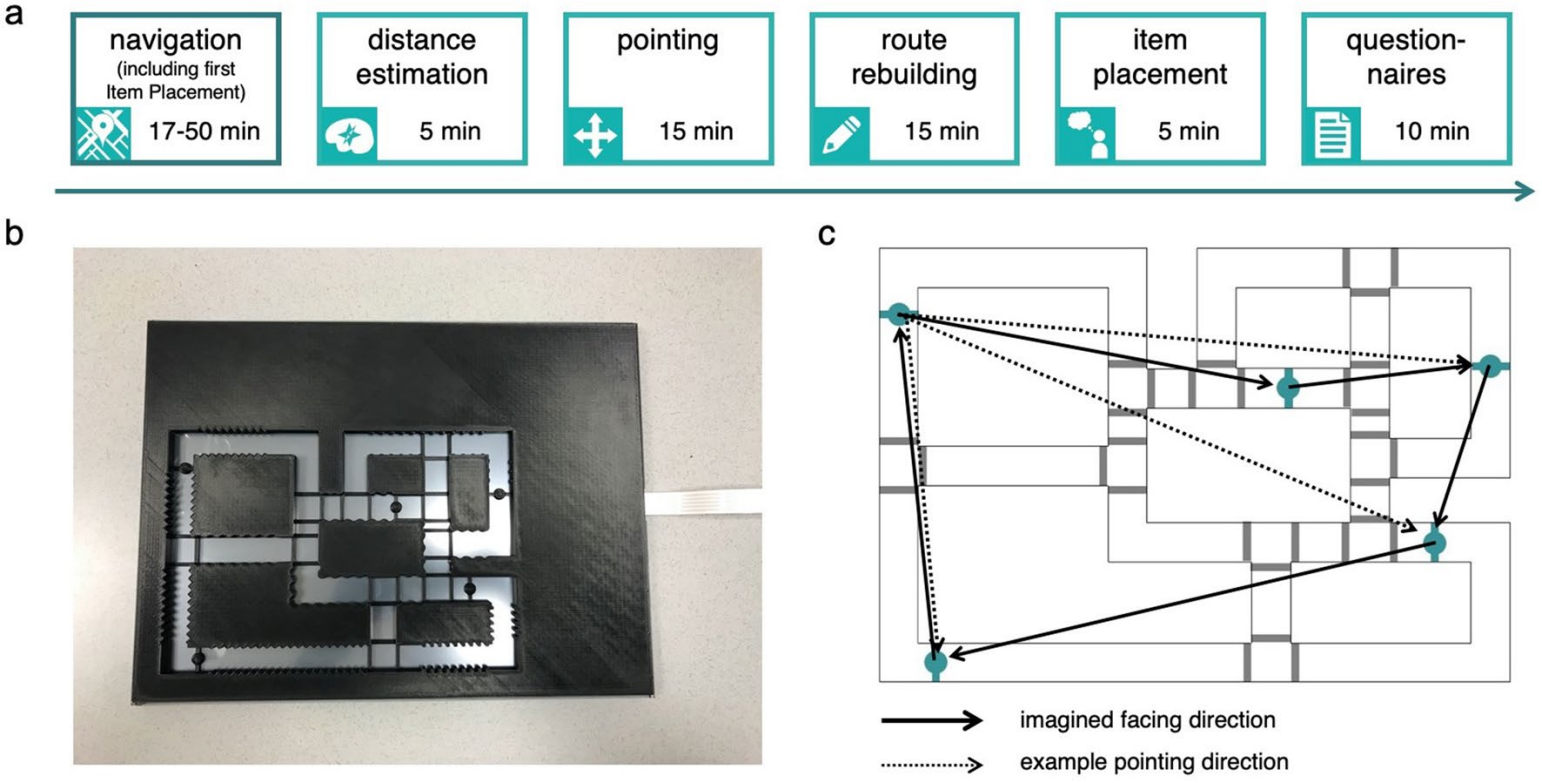
Experiment design and tactile map. (**a**) Timeline of the experiment. After general explanation of the experiment, participants started with the navigation task. This was self-paced, and completion times varied between approximately 17 and 50 min. Halfway the navigation task, participants performed an item placement task (not mentioned in the figure). After completing the navigation task, participants performed a distance estimation task (duration ~ 5 min), followed by a pointing task (duration ~ 15 min). Afterwards, they rebuilt all ten routes (duration ~ 15 min), and performed an item placement task (duration ~ 5 min). At the end of the experiment, participants filled out several questionnaires. (**b**) Tactile map on a touch panel. The streets were open, so the touch panel could track the position of the participants on the map. Small bars signaled that the participant was on an intersection. The five location markings were visible as circles on a bar on the middle of the streets. Some parts of the map had tactile textures on the edge of the surroundings that could help participants with orientation. (**c**) Schematic of the tactile map, including the imagined facing directions from each location during the pointing task (continuous arrows). Examples of pointing directions from the upper left location are also shown (dashed arrows).

During the navigation task (Fig. 1a), the participants were instructed to navigate on a tactile map by following the streets using their right index finger. It was a small-scale map (approximately 20 × 30 cm) of a city-like environment (Fig. 1b). The surroundings were 7 mm higher than the streets, and the streets had a width of 16 mm. We placed small bars around intersections like speed bumps (Fig. 1b). These were added in order for the participants to notice they arrived on an intersection and could change direction. Additionally, participants could perceive various tactile textures along the edges of the surroundings, which could facilitate orientation. On the streets of the map, five locations were marked by half spheres on the middle of the streets. During the navigation task, the participants associated these locations with different everyday items, and learned the locations of these five items. Participants heard these items as a spoken word when they arrived at the corresponding location. The items were all two-syllable nouns, and chosen such that they all occur approximately equally often in the Dutch language. This was determined using the SUBTLEX-NL database (http://crr.ugent.be/isubtlex/). The map was placed on a touch panel, and the streets were open, which allowed us to track the position of the participants on the map.

### Experimental session

At the start of the experiment, the researcher gave an overview of the session and asked the PVIs a few questions about their visual impairment: what caused the impairment, age of onset of the impairment, and a description of what they could still see. Additionally, we asked about whether they often travel autonomously in their daily life, and about their experience in O&M training and braille. After this introduction, participants performed the navigation task with the tactile map, where they learned the street layout and the locations of five items (Fig. 1a). Halfway the navigation task, the tactile map was replaced by a tactile map with the same layout, but without location markings. Here, participants had to place the five items on their remembered locations. They heard whether they were correct or not, so they could check if they were on the right track or if they had to memorise the locations better during the rest of the navigation task. After this item placement task, the original map was placed back and the rest of the navigation task was completed (Fig. 1a). Thereafter, participants performed several spatial tasks to test their knowledge of the map and the formation of a cognitive map. First, they had to estimate Euclidean and path distances between each item pair, perform a direction pointing task, rebuild routes between all item pairs using LEGO bricks, and lastly, indicate the item locations on an empty map a second time in an item placement task (Fig. 1a). These tasks are described in more detail below. At the end of the experiment, participants filled out two questionnaires: the SBSOD^49^ and the WSS^12,50^, to assess navigational abilities and general use of navigational strategies in their daily lives.

Participants sat at a table during the whole experiment, and the researcher placed the tactile map on the table in front of them. During tasks that ran on a computer (navigation task and item placement task), participants wore headphones through which they received instructions and feedback. Sighted participants and PVIs with some residual vision where blindfolded from task-relevant visual information by a curtain.

### Spatial tasks and behavioural analysis

#### Navigation task

The goal of the navigation task was for participants to learn the layout of the tactile map and the item locations by navigating from item to item. Each trial, they started on one of the five locations. They were instructed to find the shortest route to an item. When they arrived at the target location of that trial, they received feedback and heard which item was located there as a spoken word. From that location, the next trial started.

There were five items, which form 10 different pairs (or routes). The order was pseudorandomised such that participants navigated each route four times, twice in both directions (e.g., from A to B and from B to A). They therefore encountered each item 8 times. This resulted in a total of 40 routes (trials). The routes were also equally distributed across the first and second half of the task.

Participants were instructed to navigate on the map using their right index finger, to only follow the streets and not make any jumps or shortcuts. To ensure this, they were monitored by the experimenter during the task. The map was placed on a touch panel, which allowed the task script to track the position of the participant, and to detect when they arrived at the target location. To always detect this correctly, we set a small area around the location markings that counted as ‘the location is reached’. This was done in order to make sure the participant received feedback as soon as they arrived at the target location. This area was about 2 times the width of the location markings on the maps (Fig. 1b), on both sides. The task was programmed in MATLAB (MATLAB and Statistics Toolbox Release 2017b, The MathWorks, Inc., Natick, Massachusetts, United States).

Halfway the navigation task, after 20 trials, participants performed an item placement task. The tactile map was replaced by one with the same layout, but without location markings. Participants had to point out the five items on their remembered locations. They received instructions which item location they had to point out, after which they navigated there. The researcher made sure this location was recorded by the task script. Participants received feedback whether they were correct or not. The location was considered correct when it was placed within the area that counted as ‘the location is reached’ during the navigation task. When they were not precise enough, they had to navigate to the correct location first, before they received instruction to indicate the location of the next item. This task was included in order to encourage learning and recall of exact item locations. If participants were not precise enough for one or several items, they knew that they had to focus more on learning the exact item locations during the second half of the navigation task. After the item placement task, the original map was placed back, and participants completed the second half of the navigation task.

To analyse the navigation task, we computed the completion time of the two halves as well as of the whole navigation task. Additional to group differences, we tested whether there was a difference between the first and second half within the two groups. Furthermore, we computed a learning curve across trials, as the total path length navigated up until that trial, divided by the shortest route length up until that trial.

The performance on the item placement task was analysed by first computing the Euclidean distance from each placed location to its correct location. This distance error was then corrected for the maximum error possible by dividing it by the maximum distance on the map from the corresponding correct location. We calculated the mean score across the five locations, yielding one error score per participant.

#### Distance estimation task

Participants had to estimate relative Euclidean (‘as the crow flies’) and path distances of the shortest routes between each item pair. They did this on a scale from 0 to 100, where 0 would mean that two items are on the same location, and 100 was the (Euclidean or path) distance between the items that are furthest apart. The other distances had to be linearly scaled to this. The order of the item pairs was randomised for each participant. They first had to estimate the Euclidean distance between all pairs, and then the path distance. The researcher recorded the estimations on paper.

To analyse performance on the distance estimation task, we correlated the estimated distances with the correct distances, using Pearson’s correlation. This was done for the Euclidean and path distances separately.

#### Pointing task

In the pointing task, participants had to point into the direction of item locations from a particular point on the map. Specifically, they had to imagine themselves standing on one item location (standing locations), looking in a straight line at a second item location (facing location; Fig. 1c), and then point into the direction of the three other items (pointing locations) using a clockface arm. When this arm was pointing upwards (at 12 on a clock), it meant that it was pointing towards the item they were looking straight at. This was done once for each item location (with three direction estimations; see Fig. 1c for an example), resulting in a total of 15 estimations. The researcher placed the clockface arm back to upwards after each estimation. The facing locations were the same for all participants, and were determined such that they required different degrees of mental rotation (Fig. 1c). The orders of standing and pointing locations were randomised for each participant. The researcher recorded the estimations on paper, in degrees.

To analyse performance on the pointing task, we calculated the proportion of estimations that was in the correct quarter of a circle to check whether participants have a correct general feeling of direction. Additionally, we computed the mean angle error as deviation of the estimation from the correct direction (in degrees) across all pointings for each participant.

#### Route rebuilding task

Participants had to rebuild the routes between each item pair using LEGO bricks during the rebuilding task. They received an empty plate and bricks of various lengths. They were instructed to build the layout (sequence of turns along the route) as well as the distance as correctly as possible. Furthermore, they had to build the route in the direction that the researcher indicated (e.g., from item A to B, not from B to A). When the participant indicated a route was done, the researcher placed small objects at the start and end of the route and took a picture, so it would be clear during analysis from which to which location the route was supposed to go. Then the participants were given an empty plate and bricks for the next route. The order of routes was randomised for all participants.

Since participants had to build the layout as well as the distance as correctly as possible, we computed a score for both aspects to analyse performance on this task. We developed a method to score all routes as objectively as possible. This method was tested by two independent researchers, and very similar scores were obtained. The results reflect consensus of the two researchers. The complete set of rules that we used to score all routes as objectively as possible, can be found in the appendix. First, it was determined which route the participant reproduced, and this route was used as the reference for calculating the layout and distance score. In general, we calculated the layout score by counting the number of correctly built elements and dividing this by the number of elements of the reference route. An element is a part between two turns or between a turn and the beginning/end of the route. An element is correct if the preceding turn is in the correct direction. If a participant built additional elements, these were subtracted from the number of correctly built elements.

The distance score was established by calculating the proportional deviation from the correct length of each element. This was done by dividing the built length by the correct length. Only correctly built elements were considered here. The mean across elements yielded the distance score for that route.

For both scores, the mean across all ten routes was taken, yielding a layout score as well as a distance score between 0 and 1 for each participant. When participants did not build the shortest route, we first established which route they had built. This was then taken as the reference. Some of the rebuilt routes were too complicated to score only using the general methods explained above.

#### Item placement task

The item placement task at the end of the experiment was similar to the one halfway the navigation task. Participants had to indicate the location of the five items on a map without location markings. The task script instructed which item the participant had to point out, and they had to navigate there. The researcher made sure the position was recorded by the task script. Then, participants received instructions about the next item they had to point out. In contrast to the earlier item placement task, the participants did not receive feedback about whether they were correct or not. The order of item locations was randomised for each participant. We programmed the task in MATLAB (MATLAB and Statistics Toolbox Release 2017b, The MathWorks, Inc., Natick, Massachusetts, United States).

We analysed the data the same as the item placement task halfway the navigation task. The mean distance error across item locations was calculated, and we corrected this for the maximum error possible.

### Questionnaires

In order to indicate navigational abilities and general use of navigational strategies in their daily lives, all participants filled out the SBSOD^49^ and the WSS^12,50^ questionnaires. The SBSOD gave a score between 1 and 7. A higher scores indicates higher self-reported navigational abilities and sense of direction. The WSS gave a score between 1 and 5, where a lower score indicated general use of a route strategy, and a higher score general use of a survey strategy.

### Statistical analysis

Nonparametric statistical tests are appropriate, as our data has a nonparametric nature and always has values between 0 and 1 or are otherwise restricted. Therefore, when testing for significant differences between groups, we used a Wilcoxon rank sum test for independent samples. When we tested for differences within groups, we used a Wilcoxon signed rank test for paired samples. Furthermore, we correlated the scores of the SBSOD and WSS questionnaires with the performance on the spatial tasks, using Spearman’s (rank) correlation for nonparametric data. All analyses were performed in MATLAB (MATLAB and Statistics Toolbox Release 2017b, The MathWorks, Inc., Natick, Massachusetts, United States).

## Results

In short, in this study, a group of PVIs and a sighted control group learned the street layout of a tactile map, and five item locations on this map during a navigation task. Subsequently, participants performed several spatial tasks to assess cognitive map formation. First, they estimated Euclidean and path distances between each item pair, they performed a direction pointing task, then they had to rebuild all routes using LEGO bricks, and lastly they recalled the item locations. At the end of the experiment, they furthermore filled out two questionnaires that assess self-reported navigational abilities and general use of navigational strategies.

### Navigation task

**Figure 2:**
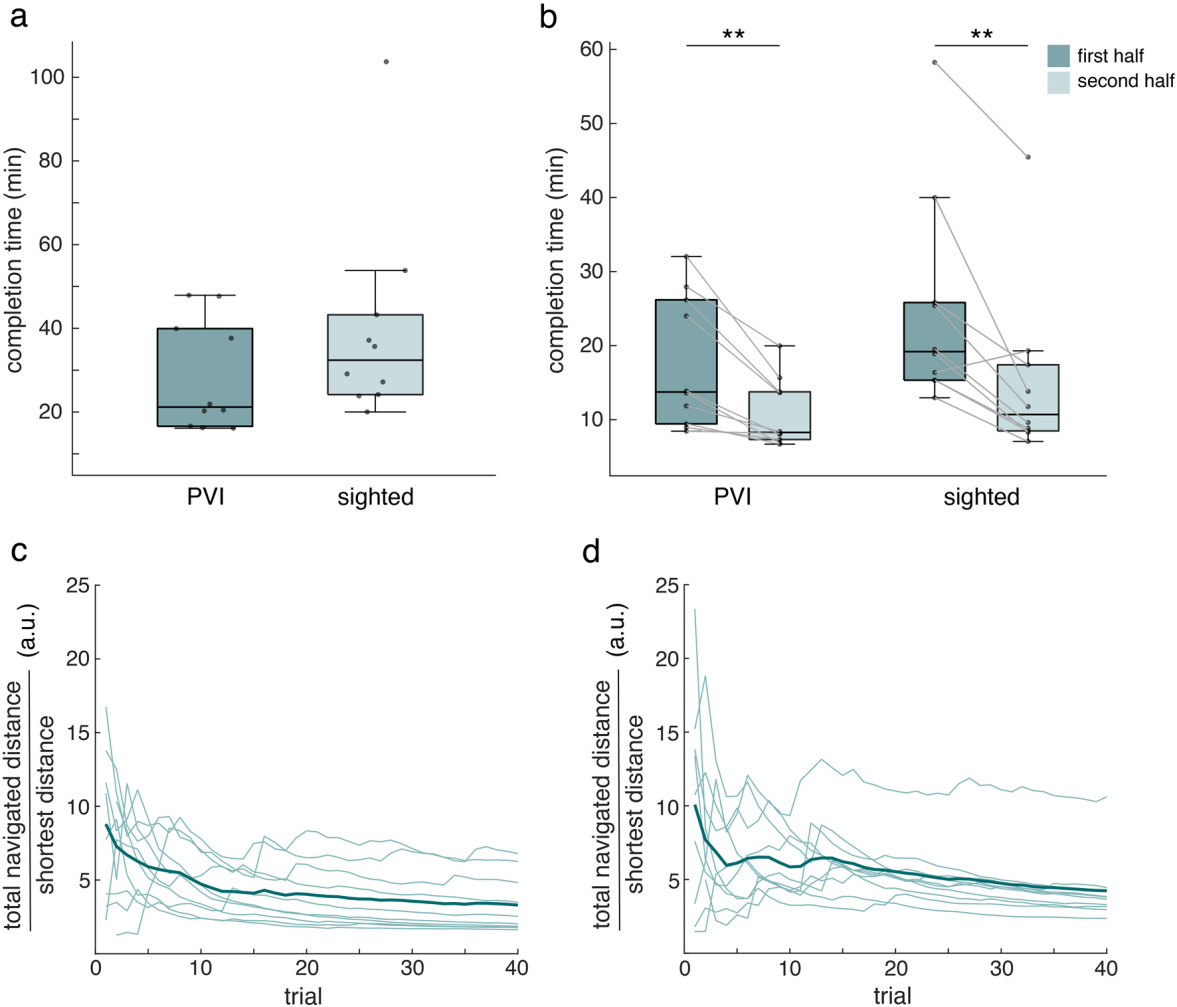
Completion times and learning curves during the navigation task. (**a**) Completion time of the whole navigation task of the PVI and sighted group (median ± 25th and 75th percentile). Grey dots indicate individual data points. (**b**) Completion time of the first and second half of navigation task of the PVI and sighted group (median ± 25th and 75th percentile). Grey lines represent individual changes in completion time from the first to second half. (**c**) Average learning curve of the PVI group. Thin lines represent individual learning curves. (**d**) Average learning curve of the sighted control group. Thin lines represent individual learning curves. **p < 0.01.

We found no significant difference between the PVI and the sighted group regarding completion time of the navigation task (Fig. 2a; Table 2). Both groups showed a significant decrease from the first to the second half of the navigation task (Fig. 2b; PVI: *p* < 0.01, W = − 55; Sighted: *p* < 0.01, W = − 47; Table 2).

**Table 2:** Test statistics W and *p*-values of all statistical tests performed on results from the spatial tasks, of the PVI and sighted group.

	*p*	W
Navigation task		
PVI versus sighted	0.212	88
First versus second half		
PVI	0.002	− 55
Sighted	0.004	− 47
Distance estimation task		
PVI versus sighted		
Eucl	0.212	88
Path	0.678	99
Eucl. versus path		
PVI	0.492	19
Sighted	0.375	− 15
Pointing task		
PVI versus sighted		
Angle error	0.571	97
Quarters	0.732	100
Route rebuilding task		
PVI versus sighted		
Layout score	0.590	79
Distance score	0.308	73.5
Item placement task		
PVI versus sighted	0.730	81
First to second task		
PVI	0.383	2
Sighted	0.109	− 22

We also computed learning curves across trials. Both groups on average showed a decreasing curve (Fig. 2c,d), which indicates that they took shorter routes across trials, suggesting learning of the map layout. There was no clear difference between the groups.

### Distance estimation task

**Figure 3:**
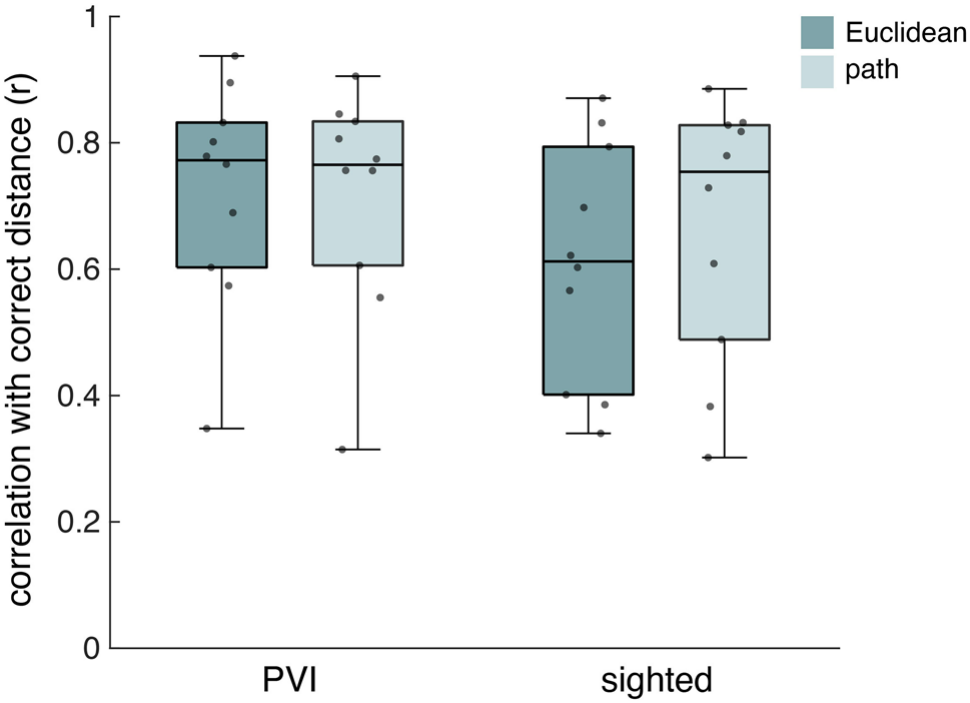
Correlation coefficients of distance estimations with correct distances. Results are shown for both groups and distance types (Euclidean and path). Individual correlation coefficients are plotted as grey dots.

On the distance estimation task, both groups showed high correlation coefficients of estimated with actual distances, for both Euclidean and path distance (Fig. 3). We did not find significant differences between groups or distance types (Table 2). This indicates good representation of distances between item locations on the tactile map. It is important to note, however, that Euclidean and path distances were highly correlated.

### Pointing task

**Figure 4:**
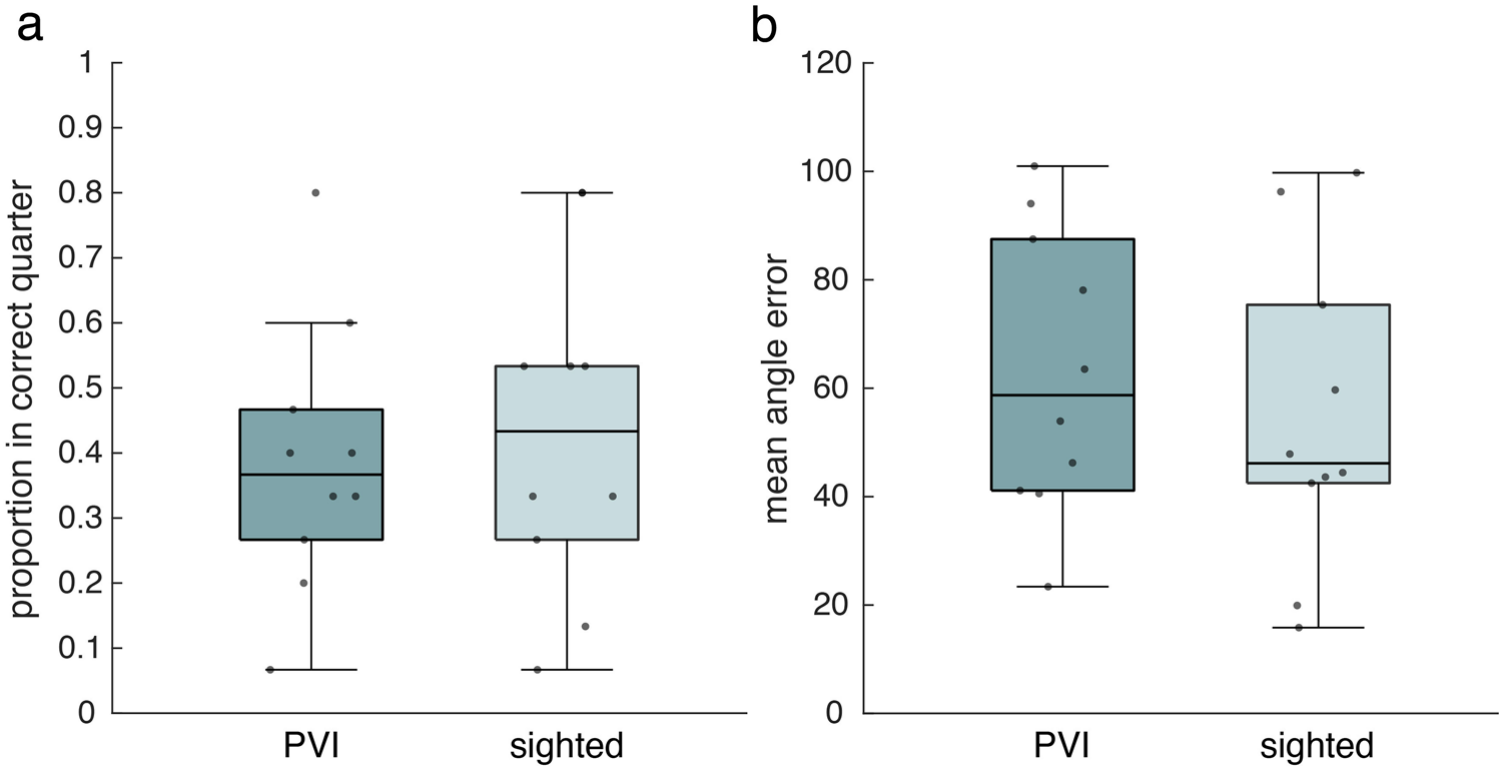
Performance on the pointing task of the PVI and sighted group. (**a**) Proportion of estimations in correct quarter of circle (median ± 25th and 75th percentile). (**b**) Mean angle error across estimations (median ± 25th and 75th percentile). Grey dots represent individual scores.

Regarding the pointing task estimations, we first looked at the proportion of estimations that was in the right quarter of a circle, to see if participants roughly knew the directions. Here, most participants had a score above chance level, which is 0.25, but only few participants had an actually good feeling of directions (Fig. 4a). We found no difference between the groups (Table 2).

When investigating the deviation from the correct direction in degrees, the mean error across all estimations was quite high in both groups (Fig. 4b). We found no difference between the PVI and sighted groups (Table 2). Results from both analyses seem to suggest that participants did not have an accurate representation of directions, or could not accurately infer this from a representation. This may have resulted from the different degrees of mental rotation they had to apply (Fig. 1c).

### Route rebuilding task

**Figure 5:**
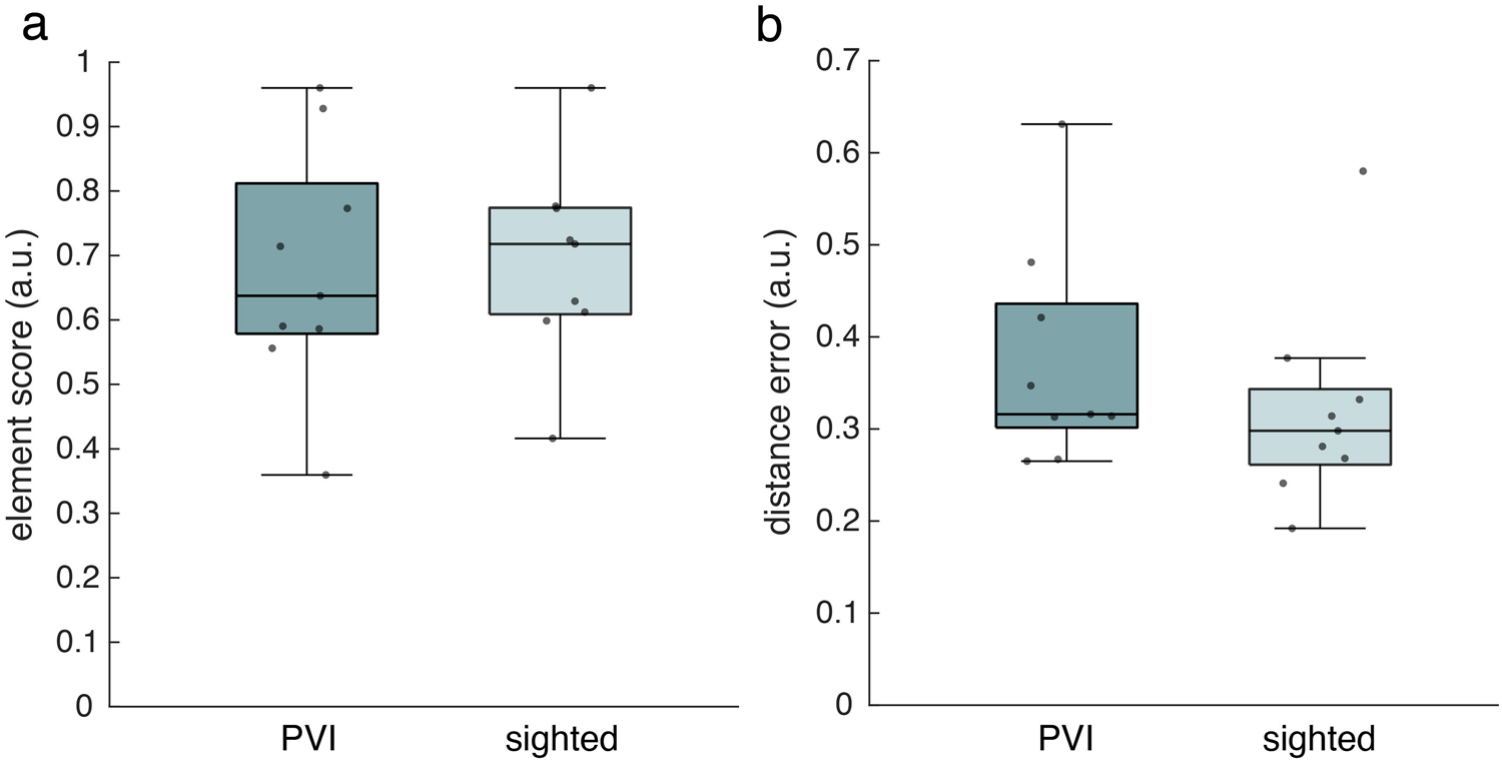
Performance on the route rebuilding task of the PVI and sighted group. (**a**) Layout scores for the route rebuilding task (median ± 25th and 75th percentile). (**b**) Distance error scores for the route rebuilding task (median ± 25th and 75th percentile). Grey dots represent individual scores.

Of the rebuilt routes, we computed a layout score and a distance error score. The PVI and the sighted group both performed acceptably considering the layout score, with a median above 60% correct route elements (Fig. 5a). Furthermore, both groups performed well regarding the distance score, with a median around 30% deviation from the correct distance (Fig. 5b). We did not find a significant difference between the PVI and sighted group for both the layout or distance score (Table 2).

### Item placement task

**Figure 6:**
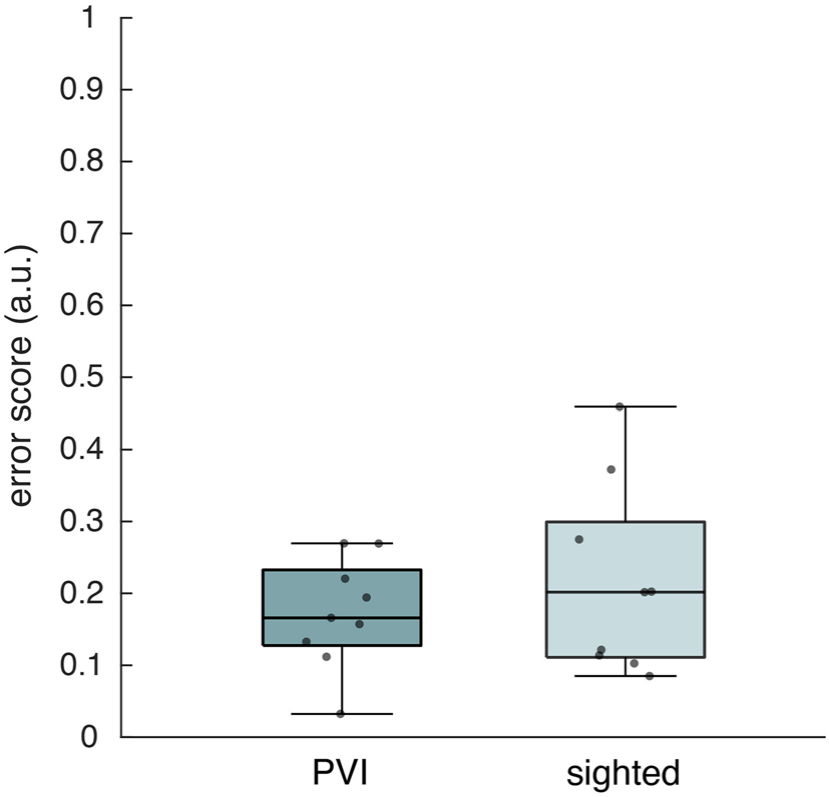
Error scores across item locations during the item placement task, corrected for maximum error possible (median ± 25th and 75th percentile). Performance of both the PVI and sighted group are shown. Grey dots represent individual scores.

On the item placement task, the PVI and sighted group obtained similar error scores, with a mean distance error under 20% of the maximum error possible (Fig. 6). Some sighted participants performed worse, but we did not find a significant difference between the groups (Table 2). One PVI did not finish this task, so also the matched control of this person was taken out of the analysis.

Participants also performed an item placement task halfway the navigation task. When comparing those scores with the task at the end, most participants showed a decrease in error score, hence an improvement in performance. This improvement, however, was not significant in both the PVI and sighted group (Table 2). This lack of significance is probably driven by participants who performed already very well during the first item placement task and showed a similar or slightly decreased performance during the second.

### Role of navigational strategies

At the end of the experiment, participants filled out the SBSOD^49^ and WSS^12,50^ questionnaires. The distribution of SBSOD-scores across all participants was at the higher end. This indicates that our group of participants have relatively high self-reported general navigational abilities and sense of direction. The distribution of WSS-scores across all participants was around the middle region. This indicates that most participants in this study do not have a strong tendency towards a route or a survey strategy, but rather sometimes use a route strategy and sometimes use a survey strategy. For both questionnaires we did not find differences between the PVI and the sighted control group.

To investigate whether there is a relationship between the self-reported abilities and the performances during our experiment, we correlated the scores on the questionnaires with the results of the spatial tasks. First of all, the SBSOD correlated negatively with pointing task angle error scores across all participants (r = − 0.52, *p* < 0.05) and in the PVI group (r = − 0.85, *p* < 0.01). The same scores in the PVI group also correlated negatively with WSS (r = − 0.66, *p* < 0.05). For the rebuilding task, we found a significant negative correlation of distance error scores with the SBSOD in the PVI group (r = − 0.72, *p* < 0.05), but not with the WSS. We did not find significant correlations of performance on the distance estimation or item placement task with either of the questionnaires. The correlations that we found are in the expected directions, since a higher score on the questionnaire means relatively good navigation and orientation abilities. The correlations seem to show that these relate to better performance on the spatial tasks.

Additionally, we tested for effects on task performance of experience in autonomous navigation, O&M training and braille across PVIs, and of education level, age and gender across all participants. We found no effects of education level, age or gender, nor an indication for influence of autonomous navigation in daily life or O&M training experience. With braille experience, we found a shorter completion time on the navigation task (*p* < 0.05, W = 23), lower angle error on the pointing task (*p* < 0.01, W = 21), and a lower distance error score on the route rebuilding task (*p* < 0.05, W = 15). No effects on other spatial performances were found. We did find higher SBSOD-scores with braille experience (*p* < 0.05, W = 12), but no relationship with WSS-scores (*p* = 0.133). In addition, PVIs with braille experience completed the navigation task faster than sighted participants (*p* < 0.05, W = 30), but no other significant differences between PVIs with braille experience and sighted participants were discovered.

## Discussion

We assessed formation of a cognitive map of a small-scale tactile city-like environment in persons with a visual impairment and sighted control participants. To this end, participants learned the street layout of a tactile map, and five item locations. Thereafter, they estimated distances and directions between item locations, reproduced routes between them, and recalled their exact locations. Additionally, we related the performance on these tasks to their general navigational abilities and strategies using two questionnaires.

### Cognitive map formation by PVIs and sighted persons

In general, the participants performed well on the spatial tasks. Considering the navigation task, the reduction in learning time from the first to the second half, and the decreasing learning curves indicate learning of the tactile map. It shows that participants found shorter routes between item locations. No differences were found between the PVIs and the sighted group, suggesting equal map learning rates in the groups. The improvement from the first to the second item placement task suggests further learning of the spatial layout.

In the distance estimation task, we found strong correlations of estimated distances with correct distances, indicating good representation of relative distances between item locations in a cognitive map. Euclidean distance were not explicitly learned during the navigation task. Therefore, an offline representation of the map is most likely required to infer this information. We did not find differences between PVI and sighted participants, suggesting equally accurate distance representations. Our findings are supported by research showing that sighted persons can accurately estimate distances between locations in a virtual environment, and that they are represented in the hippocampus^7^. Furthermore, multiple studies suggest that PVIs and sighted persons perform equally well on estimating distances^20,52^. Other research indicates that PVIs perform well on ordering routes from shortest to longest after exploring a tactile city map^53^. Nonetheless, it seems that early blind persons generally overestimate distances more than late blind persons^54^, while sighted persons show an underestimation^55^. Not all research support the equal representation of distances in PVIs and sighted persons^13,21,30–32^. Sighted and late blind persons showed good representation of distances between tactilely presented object locations, but early blind persons did not^31^. This was measured by correlating the mental route scanning time with actual distance. It could be that mental route scanning is more difficult without visual experience. Another speculation is that our visually impaired participants, who were mostly early blind, performed well on this task because they are generally relatively good navigators, as indicated by the questionnaires. Nevertheless, it is difficult to specify whether results from other studies, showing worse performance by PVIs compared to sighted participants^13,21,30–32^, are influenced by including worse navigators, because general navigation abilities are often not measured.

Participants in our study did not perform particularly well on the pointing task. They did perform above chance level, but the angle error was quite high in both groups. Nonetheless, the results indicate that PVIs and sighted participants had an equally correct feeling of directions between item locations.One consideration here is that this was the only task that required mental rotation. All other tasks, including distance estimation, could have been done from the perspective of the own body. The mental rotation could have made the pointing task harder to execute^21,32^. Similar results were found in previous research^32^. Here, the requirement of mental rotation also posed difficulties in accurately estimating angles. They did, however, show a significantly better performance in sighted persons than PVIs^32^. Furthermore, it has been suggested that it is harder to represent directions in a cognitive map after only walking a route compared to a combination of a tactile map and direct experience^25^. This highlights the usefulness of a tactile map in building up a cognitive map of an environment.

The results from the rebuilding task indicate that the participants in the PVI as well as the sighted group could derive routes between the item locations from their cognitive maps equally well. Despite variations between individuals, our participants got acceptible layout scores and good distance error scores. This indicates overall quite good knowledge of the map and routes between the item locations. The ability of PVIs to reproduce maps or routes after exploring an audio-tactile map is supported by previous work^27,34^. Similar results have been found in PVIs after learning an auditory environment^56^. The quite good layout scores of the route rebuilding task indicate good route knowledge of the map in both groups^57^. In general, good feeling of distance would suggest survey knowledge^12,57^. Therefore, our participants seem to have good route as well as survey knowledge of the tactile map. One thing to consider, however, is that no scaling was necessary to rebuild the routes. We speculate that to correctly reproduce distances (length of paths between turns) during the route rebuilding task, using information from relative positions of the hand to the body or remembering a sequence of distances might have been sufficient. Route knowledge might therefore have sufficed to perform well on this aspect.

On the last spatial task, the item placement task, the scores were high, with most participants having an error score below 0.2, which can be seen as deviation of less than 20% of maximum error. This suggests that most participants had the item locations correctly stored in their cognitive map. We found no difference in performance between the PVIs and sighted participants. In contrast, a previous study found that congenitally blind participants were worse at judging statements about spatial relationships like relative positions than sighted participants^30^. The objects in their experiment, however, were not placed on a tactile framework like our tactile map, which could have made it more difficult to represent position of objects compared to each other. These findings may also signal a difficulty congenitally blind persons experience in acquiring survey representations^30^. In our study, a survey representation is presumably not required to represent the item locations in a cognitive map.

Taken together, our findings indicate that both the PVI and sighted control group were able to build up a cognitive map of the tactile environment. We did not find differences in accuracy between the groups. It could be that our participants all are generally good navigators and therefore perform similarly, however, differences might arise with a larger sample size. With respect to differences in spatial abilities between PVIs and sighted persons, there are many discrepancies in the literature. Some studies suggest that PVIs perform worse on spatial tasks than sighted persons^13,20,32^. There is also research, however, that shows that PVIs perform similar^56,58,59^ or even better^13,21,46,60^ than sighted persons considering spatial cognition. Furthermore, some studies suggest that there are differences between PVIs who became blind very early or later in life^21,30,31^. What gives rise to these discrepancies is unclear until now. We speculate that it might be partially caused by how exactly the experiments and spatial information are presented to participants, as they might be (unintentionally) in favour of PVIs or sighted persons. For example, it could be that visual experience has an advantage in how a task is instructed. Contrarily, we consider that completely blindfolding a sighted person may give rise to a situation that is uncomfortable and highly unusual for this person but not for a PVI, and may affect task performance. In our study, we found that PVIs performed equally well as sighted persons. Most of our visually impaired participants had experience with traveling, reflected in their willingness to travel to the institute to participate in our experiment. They might furthermore be less afraid because they have relatively good navigational abilities, which is also reflected in questionnaire scores. This may have affected performance on the spatial tasks. To the contrary, some PVIs had little O&M experience and did not travel autonomously. This did, however, not affect performance, indicating that even with less experience, PVIs can build a fairly accurate cognitive map. It is important to note, however, that our sample was not balanced or large enough regarding autonomous navigation and O&M experience to draw any conclusions with confidence. Furthermore, we found no effects of age on any task performance across all participants. Although many studies suggest that navigational abilities decreases with age^61–64^, there are a few explanations why we did not observe that effect. For instance, most of our participants were around 60–70, therefore, the dispersion of ages might not be sufficient to detect a decline due to age, which often arises around the age of 60^61–64^. Nevertheless, most of our participants were generally good navigators, hence age might have had a small effect on their navigation abilities. Taken together, we speculate that effects of age on spatial task performance were limited in the current study. We furthermore found lower angle errors on the pointing task, lower distance errors on the rebuilding task, and higher SBSOD-scores in PVIs with braille experience compared to PVIs who do not read braille. We speculate that this was not due to braille experience itself, but rather because our PVIs with braille experience were coincidentally slightly better navigators and had better sense of direction, as measured using the SBSOD. Higher SBSOD-scores have been linked to better performance on the pointing and rebuilding tasks, and better survey knowledge in the rest of our data as well (see “Role of navigational strategiesandperspectives” section). The coincidence might have appeared because of the low sample size (6 PVIs with and 4 PVIs without braille experience). The higher navigation abilities could have been reflected in better performance on the pointing task and route rebuilding task in these participants. Our speculation is supported by findings that PVIs with braille experience did not perform better nor had a higher SBSOD-score than sighted participants (who did not have braille experience). Nevertheless, PVIs with braille experience completed the navigation task faster than sighted and PVIs without braille experience. This effect diminishes when only considering completion time of the second half of the navigation task. Therefore, we think that the initial difference may arise because braille readers had more experience in processing tactile stimuli. Taken together, we speculate that braille experience itself did not have an effect on cognitive map formation. Important to note, however, is that our sample size is too low to confidently compare PVIs with and without braille experience and draw strong conclusions on this matter.

Our finding that PVIs and sighted participants form equally accurate cognitive maps, is furthermore in line with a growing body of evidence suggesting modality-independent representation of space^65–70^. This theory implies that spatial information gathered through different sensory modalities contribute to one amodal spatial representation, rather than a distinct representation for each modality^65–68^. Cognitive maps would therefore be similar in PVIs and sighted persons, supporting functionally similar spatial behaviour^68–70^. We show this similarity in our data, which is therefore an important finding contributing to the theory of amodal representations of space.

Although we did not find differences between PVIs and sighted persons, the results show a large spread of performance within groups in all tasks. The spread may have been facilitated by the fact that the tactile environment was not memorised at ceiling during the navigation task. This was done on purpose, in order for differences to arise amongst people, for instance between good and poor navigators. The spread may furthermore reflect heterogeneity of spatial abilities within the groups, and may relate to the outcomes of the SBSOD and WSS questionnaires. Here we found that most participants are generally good navigators, but there is variation nonetheless. The WSS indicated no clear tendency towards a route or survey strategy. Most participants may sometimes use a route, and sometimes a survey strategy. To better disentangle this, it might be helpful to beforehand include people who are very good navigators, and others who are poor navigators. Nevertheless, the correlations between the questionnaires and tasks might suggest that there is a relationship, and this might become clearer if we can compare extremes (very good vs very poor navigators).

### Role of navigational strategies and perspectives

In addition to cognitive map formation in PVIs, we wanted to investigate the relationship between general use of route and survey strategies and performance on the spatial tasks. We did not find differences in performance between PVIs and sighted persons. Nonetheless, the fact that we do find some significant relationships between the questionnaires and task performance, indicates that the tasks might be sensitive enough to show differences if there were any. We found a negative correlation of SBSOD-scores with angle errors of the pointing task, and distance errors of the rebuilding task. This suggests that better general navigational abilities relate to better performance on these tasks. More specifically, these tasks both represent survey-type knowledge, therefore, our findings indicate that better navigational abilities relate to better survey knowledge. We found the same relationships of task performance with WSS-scores. This suggests that a tendency to use survey knowledge is related to better survey-type knowledge. Such relationships between spatial task performance and strategy is supported by previous findings. For instance, people who used a survey strategy were better at reproducing directions between locations during a pointing than people who used a route or verbal strategy, especially with higher degrees of mental rotation^32^. Furthermore, participants who used allocentric strategies performed better on estimating distances between tactile objects than participants who used egocentric strategies^35^. Nevertheless, in the current study, when looking as WSS-scores only, we did not find a clear distinction between persons who mostly use a route strategy or a survey strategy. To be able to disentangle this, it might be helpful to beforehand include people based on their preferred strategy.

In our study, we found that PVIs and sighted persons generally use similar types of navigational strategies. When looking at previous research, there are a lot of discrepancies. There is a lot of literature indicating that PVIs in general are less capable, or have a much lower tendency to use a survey strategy, and mostly, if not only, use a route strategy^20,30,71^. This might be the main reason why those people would perform less well on spatial tasks. Other papers, however, state that PVIs tend to use a route strategy because they are offered very few opportunities to employ a survey strategy^35,44,48,72^. These studies suggest that PVIs can adopt a survey strategy and perform equally well as sighted persons if the required spatial information is made available. In our study, most participants, both PVIs and sighted, have high self-reported general navigational abilities, and tend to use similar navigational strategies. Our results also indicate that they construct an equally accurate cognitive map, and acquire an equal level or survey knowledge. Therefore, our results support arguments that PVIs have similar spatial cognitive abilities as sighted persons when they get the chance to use similar strategies^35,44,48,72^. We suggest this for our group of generally good navigators, but it is unclear whether this would also be the case for poor navigators. We cannot elaborate on this based on our current data. Therefore, it would be relevant to explore this further in future research, for instance by selecting participants up front based on good or poor navigation abilities.

### Relevance and future directions

Our findings indicate that PVIs and sighted persons can build up an equally accurate representation of a tactilely presented environment. We furthermore show that in our tasks, PVIs acquired survey knowledge similarly to sighted persons. A survey strategy is more flexible than a route strategy, as it allows for example for computation of detours when the usual route is partly blocked. Therefore, having survey information available could be beneficial for wayfinding in PVIs. This may not be highly relevant when having to navigate a route only once, but it may be when they have to find their way in an environment that they will encounter more often. Then it would be advantageous to build up a survey representation of the environment.

One limitation of presenting an environment as a tactile map, is that it is always learned from one perspective. Participants always perceive the environment from the same side (i.e., they always face North). This might make the spatial representation less flexible than when it is perceived from first-person perspective. In addition, a cognitive map may be more robust when a person can integrate spatial information from multiple sensory modalities, such as proprioceptive and auditory information. Therefore, one has to be careful when generalising our findings to real-world navigation. However, purely tactile information seems to be sufficient to build up some sort of cognitive map. Furthermore, visual experience may be of influence on cognitive map formation. In our study we did not find differences between persons who acquired blindness early or late in life, but our sample size was too small to draw any conclusions.

To further examine distinctions between good and poor navigators, or between the use of route and survey strategies, one could perform further experiments where participants are selected beforehand on navigational abilities or strategy use. Furthermore, since we behaviourally show that PVIs can build up representations of a tactile map, a follow-up study could examine these representations in the brain^7,8^. For example, one could analyse neural representations of learned locations in a tactile environment, and investigate whether spatial information such as distance is reflected in these representations.

## Conclusion

In our study, we show that persons with a visual impairment and sighted persons can build up a cognitive map of a tactilely presented environment equally well. They perform similarly on spatial tasks that require route as well as survey knowledge. We therefore also suggest that PVIs are not only able to employ a route strategy, but also a survey strategy if they have the opportunity to access route-like as well as map-like information such as on a tactile map.

## Data availability statement

The data and all materials for the experiments reported here are available. Access to the data can be requested by contacting the corresponding author.

## Supplementary Information

Supplementary Information.
