# Supplementary material for: Cognitive map formation through tactile map navigation in visually impaired and sighted persons
Source: Sci Rep. 2022 Jul 7;12:11567. doi: 10.1038/s41598-022-15858-4 (PMC9262941; doi:10.1038/s41598-022-15858-4)
Supplement: Supplementary file 1 — Supplementary Information. [file 41598_2022_15858_MOESM1_ESM.docx]

**Supplemental information**

For scoring the rebuilding task, a method was developed to score all routes as objectively as possible. First, it was determined which route the participant reproduced, and this route was used as the reference for calculating the layout and distance score (see Figure S1a for the reference map). In general, the layout score was calculated by counting the number of correctly built elements (an element is a part between two turns or between a turn and the beginning/end of the route) and dividing this by the number of elements of the reference route (see Figure S1b and c for an example). An element is correct if the preceding turn is in the correct direction. If a participant built additional (extra) elements, these were subtracted from the number of correctly built elements. Additionally, we applied the following rules:

1. The routes were always thought of as from item 1 to item 2 as they were instructed to the participant. Exception: point 2 and 3.
2. When the beginning and end of a route are clearly correct, but the participant built too many or too few elements in the middle, the beginning and end are considered correct, and the extra or missing elements in the middle a subtracted from the number of correctly built elements (see Figure S1c for an example).
3. The other way around as point 2, when only a part of a route is built, and this is most likely the middle part of a reference route, the built elements are still considered to be correctly built elements.
4. If a turn is in the wrong direction, the following element is marked as incorrect, but the elements after that are scored as usual. Exception: point 5.
5. When the turn after an incorrect turn is in the wrong direction again, but it is in the correct direction to get to the target location when viewed from the participant’s location, this is not marked as incorrect again, because the participant most likely wanted to get back into the direction of the target location (see Figure S1d for an example).
6. If an element has a small kink (see Figure S1e), this was considered and accidental turn and counted as -1 correctly built elements (instead of -2 for two incorrect extra elements).
7. When the researcher can’t make anything of the rebuilt route, it is given a layout score of zero, and the corresponding distance score is left empty.

The distance score was established by calculating the proportional deviation from the correct length of each element. This was done by dividing the built length by the correct length. Only correctly built elements were considered here. The mean across elements yielded the distance score for that route (see Figure S2a for an example). Additionally, we applied the following rules:

1. When a participant built a very long element, which most likely misses some turns, it is considered that the participant still wanted to reflect the distance it spanned, so the total distance of those missing elements was taken as the reference distance (see Figure S2b for an example).
2. In case of an ‘accidental kink’ (see layout score point 6), the length of the total element (disregarding the kink) is considered the built length.
3. When an element is marked as incorrect because the preceding turn was in the wrong direction (see rules for layout score), this element is still considered for the distance score.
4. When some space was left between bricks, this space was considered as filled, to get a continuous route.


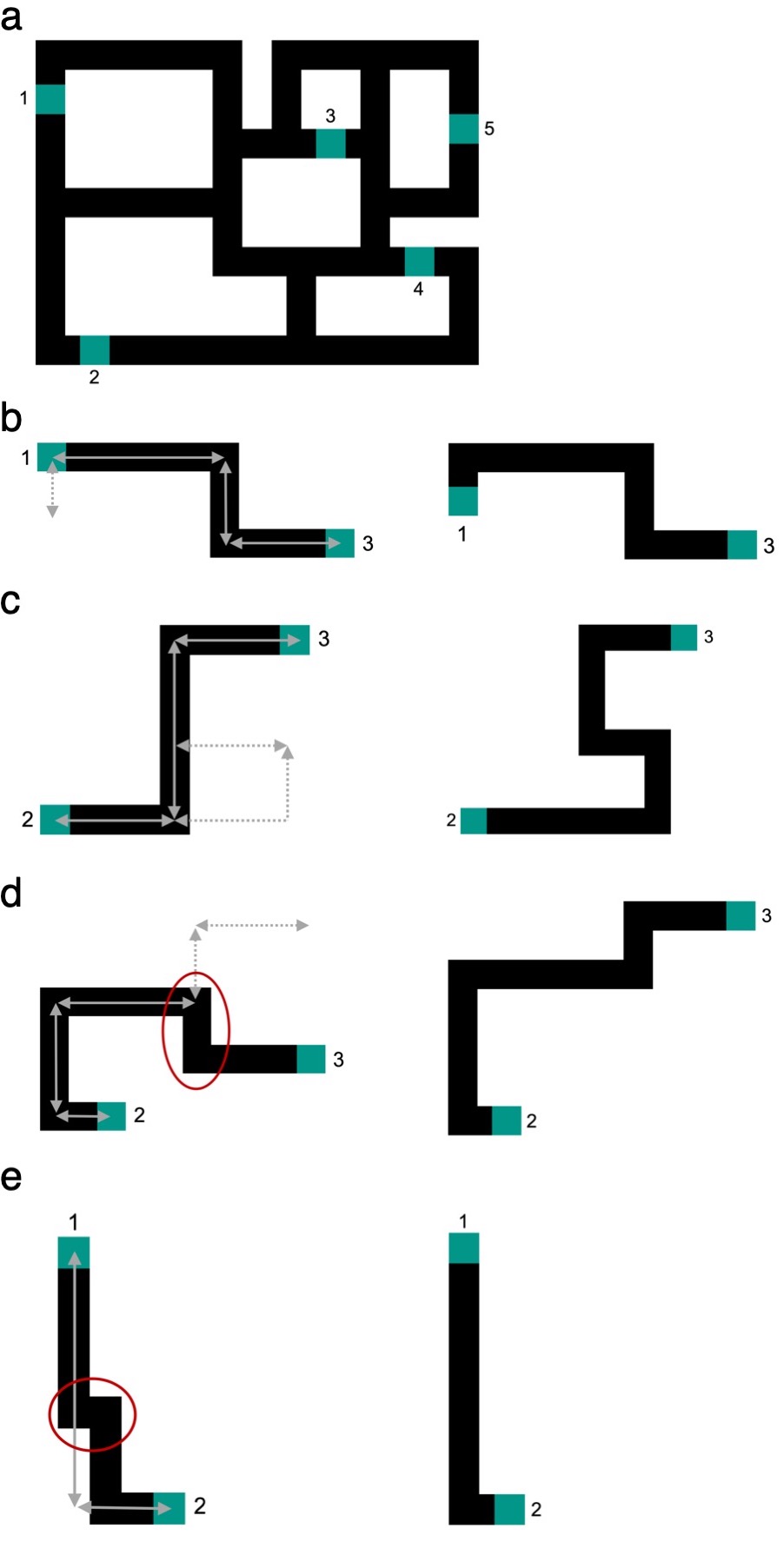


**Figure S1**. Examples of layout score calculations of the rebuilding task. Images are standardised with black paths and green locations. In (**b-e**), rebuilt routes are shown on the left, and reference routes on the right. Continuous grey arrows are correct elements, dashed arrows are missing elements compared to the reference route. (**a**) Total reference map including five locations. (**b**) The reference route has 4 elements. The rebuilt route has 1 missing element, so the layout score is (4-1)/4 = 3/4. (**c**) The reference route has 5 elements. The beginning and end of the rebuilt route seem correct, but 2 elements in the middle are missing. The layout score is (5-2)/5 = 3/5. (**d**) The reference route has 5 elements. One turn in the rebuilt route (red circle) is in the wrong direction compared to the reference route. Only the first turn (and thus one element) is considered incorrect. The layout score is (5-1)/5 = 4/5. (**e**) The reference route has 2 elements. The long element in the rebuilt route contains a small kink (red circle). This counts as 1 minus point, so the layout score is (2-1)/2 = 1/2.


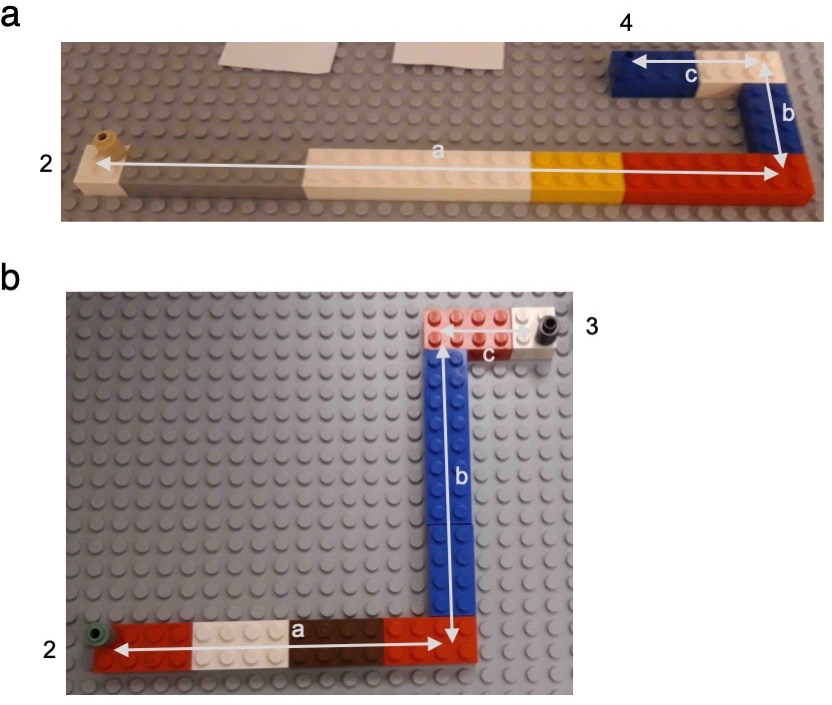


**Figure S2**. Examples of distance score calculations of the rebuilding task. Shown are two examples of actually built routes using LEGO bricks. The location numbers are indicated. Grey arrows are correct elements.

(**a**) 3 out of 3 elements are built correctly. The proportional deviation from the reference length is calculated for each element:

Element a: correct length = 27, built length = 32 → 32/27 = 1.19 → deviation from 1 is 0.19

Element b: correct length = 8, built length = 8 → 8/8 = 1 → deviation from 1 is 0

Element c: correct length = 5, built length = 8 → 8/5 = 1.6 → deviation from 1 is 0.6

Distance score for this route is (0.19 + 0 + 0.6)/3 = 0.26.

(**b**) 3 elements out of 5 are built. The beginning and end of the rebuilt route seem correct, but 2 elements in the middle are missing (see Figure S1.C). The length of b is considered as spanning the missing part as well. The proportional deviation from the reference length is calculated for each element:

Element a: correct length = 16, built length = 16 → 16/16 = 1 → deviation from 1 is 0

Element b: correct length = 16, built length = 16 → 16/16 = 1 → deviation from 1 is 0

Element c: correct length = 8, built length = 6 → 6/8 = 0.75 → deviation from 1 is 0.25

Distance score for this route is (0 + 0 + 0.25)/3 = 0.08.
